# Can active music making promote health and well-being in older citizens? Findings of the music for life project

**DOI:** 10.1080/17571472.2016.1152099

**Published:** 2016-03-11

**Authors:** Susan Hallam, Andrea Creech

**Affiliations:** ^a^ Department of Lifelong and Comparative Education, UCL Institute of Education, London, UK

**Keywords:** Music making, public health, ageing population, well-being, cognition

## Abstract

Although there is now an accepted need for initiatives that support older people’s well-being, little attention has been paid to the potential for music making to effect a significant contribution to the quality of life of older people. The research summarised here explored the role of music in older people’s lives and how participation in community music making can enhance their social, emotional and cognitive well-being. The research comprised three UK case study sites, each offering a variety of musical activities. At each site, a sample of people aged 50+ (total *N* = 398), some of whom had recently begun musical activities and others who were more experienced, were recruited to complete questionnaires that assessed quality of life. A control group (*N* = 102) completed the same measures. In-depth interviews were carried out with a representative sample, followed by observations of musical activities, focus groups and interviews with the facilitators of the activities. Higher scores on the quality of life measures were found consistently among the music participants, in comparison with the control group with ongoing benefits into the 4th age. Analysis of the qualitative data demonstrated: (1) *cognitive benefits* including challenge, the acquisition of new skills, a sense of achievement, and improvements in concentration and memory; (2) *health benefits* including increased vitality, improved mental health and mobility and feelings of rejuvenation; and (3) *emotional benefits* including protection against stress, protection against depression, support following bereavement, a sense of purpose, positive feelings, confidence and opportunities for creativity. Participants also identified a number of barriers to participation including lack of information about opportunities for making music. Ways that GP surgeries might support participation in music making are considered.

## Why this matters to us

Historically, there has been much research on the wider benefits of music for children and young people. It is only recently that there has been interest in exploring whether music could offer benefits to older people. Having demonstrated that there are enormous benefits to older people in relation to their well-being including social, cognitive, emotional and health benefits, we wish to disseminate the findings widely and try to ensure that there are opportunities within the community for older people to participate in organised music making. These opportunities need to be available locally to minimise travel, in venues which are accessible for those who may have physical disabilities, with opportunities for public performances. The activities also need to be supported by high quality facilitators.

## Key messages

• Participating in group music making can contribute to the quality of life of older people.
• The research reported here showed that those actively participating in making music had consistently higher measures of well-being than those participating in other social group activities which continued into the 4th age.
• Participants reported social, cognitive, emotional and health benefits of participation in music.
• Participants reported difficulties in accessing information about opportunities for making music. GP practices may be able to provide such information.

## Introduction

The developed world is currently facing a major demographic transition. In the UK, the number of people over 65 is projected to reach 21.3 million by 2071, those over 80 reaching 9.5 million [1] with an estimate of 64,000 expected to reach the age of 100 by 2033.[2] These changes present considerable economic challenges to policy-makers as they attempt to meet the ever-increasing demand for support for the elderly. As a result, ways to maintain well-being and the good health of the ageing population for as long as possible are becoming increasingly important. The research reported here explores the possibility that active engagement with making music may contribute to this agenda.

There is increasingly compelling evidence that active music making can impact on physical and psychological health and a range of cognitive functions.[3,4] Participation in a wide range of musical activities has been shown to provide a source of enhanced social cohesion, enjoyment, personal development and empowerment for older people supporting collaborative learning, friendship, a sense of belonging, enhanced subjective wellbeing and access to new social roles and relationships.[5] Music can also provide a sense of contentment, satisfaction and peace.[6] Choir participants have commented that singing stimulates cognitive capacity including attention, concentration, memory and learning [7] and similar benefits have been found with older adults receiving individual piano lessons over a six-month period.[8] While active engagement with making music cannot prevent dementia, there is some evidence that it can reduce the risk [9] and there is evidence that those engaged in music making have fewer health issues, fewer falls, fewer doctor’s visits and use medication less than control groups.[10,11] Overall, there is an increasing body of evidence that actively engaging in making music has benefits for older adults in terms of their health, psychological well-being and cognitive functions. The Music for Life Project aimed to add to that literature exploring the ways in which participating in creative music making could enhance the lives of older people in England through its influence on their social, emotional and cognitive well-being while also examining the specific process through which any such impact occurred.

## Methods

Three case study sites acted as partners in the research: The Sage, Gateshead; Westminster Adult Education Service; and the Connect Programme at the Guildhall School of Music. This resulted in four cohorts:

• The Silver Programme at the Sage Gateshead provided a wide range of musical opportunities for people over the age of 50 including singing of many kinds, the playing of steel pans, guitars, ukulele and recorder, and activities involving folk ensemble, music theory and samba. The programme aimed to develop the current and past skills of participants as well as encouraging and developing new and emerging ones, enhancing musical abilities and encouraging positive mental and physical heath. Participants had the opportunity to perform regularly in public concerts.
• The Music Department of the Westminster Adult Education Service (WAES) ran a wide range of musical programmes in a range of musical genres, specialising in singing, playing instruments, sound engineering and using sequencers, music theory and composing.
• The Connect Programme of the Guildhall School of Music and Drama ran community projects with people of all ages in East London. The projects were distinctive in that their focus was on activities where participants created and performed music together, linking storytelling and reminiscing to creative music-making. The musical activities with older people took place in the community rooms of sheltered housing accommodation in East London. The activities included intergenerational music sessions involving older people making music with children from local primary schools.
• A control group was made up of individuals attending language classes provided by the Westminster Adult Education Service (4 groups) and convenient sites in other places including art/craft classes (5 groups); yoga; social support (2 groups); a book group; and a social club.

## Research methods

The research was undertaken over nine months using a variety of methods including:

For the three music cohorts:

• individual interviews with music participants (*n* = 30);
• focus group interviews with music participants (15 focus group interviews);
• videos and observations of music sessions (45 videos, notes made of 25 sessions);
• videos and observations of musical performances (3); and
• interviews with music facilitators (12).

For all four cohorts, including the control cohort:

• Questionnaires which were completed by participants in control and music cohorts at the start of the research and again nine months later. 345 members of the music groups completed questionnaires at the start of the research, 147 at the end. 89 members of control groups completed questionnaires at the start of the research and 36 at the end. The questionnaires included the CASP-12 measure of quality of life and the Basic Psychological Needs Scale. The CASP-12 measure [12] including subscales relating to control, autonomy, self-realisation and pleasure. Deci and Ryan’s [13,14] Basic Psychological Needs Scale comprising 21 items organised into subscales for control, autonomy and relatedness. These two scales provided a robust measure of quality of life, focusing on cognitive, emotional and social well-being.

## The sample

The age range of the sample was 50–93 with 246 members of the music group in the Third Age (50–75) and 92 in the Fourth Age (over 75) (60 did not state their age). The majority had been involved in professional occupations. Three quarters had some kind of prior musical experiences and could read music at a basic level but many classed themselves as beginners. Only 4% described themselves as ‘very good’, while the remainder described themselves as either average or good.

## Ethics

Ethical approval was sought from and granted by the Ethics Committee of the UCL Institute of Education.

## Findings

### Questionnaire replies

Statistical analyses showed significant differences with regard to scores on the CASP-12 and Psychological Needs measures between those participating in the musical and non-musical groups at the start of the research with consistently, more positive responses from the musical groups. A factor analysis was undertaken using the items from the CASP-12 and the Basic Psychological Needs Scale to assess the extent of conceptual overlap between the two measures. This produced three overlapping factors. The first related to having a positive outlook on life (purpose), the second to lack of autonomy and control (autonomy/ control), and the third to positive social relationships, competence and a sense of recognised accomplishment (social affirmation). Comparisons of those engaged in music making with those participating in other activities at the start of the research revealed statistically significant differences on all three factors with the music groups having more positive responses. Table 1 sets out the factor scores.

**Table 1.** Comparison of factor scores between those participating in music and non-music groups.

	Music (*n* = 280)	Non Music (*n* = 62)		Effect size
Factor 1: Purpose	0.088	–0.398	*F* = 12.39 (1,340) *p* = .0001	.19
Factor 2: Autonomy/control	–0.068	0.310	*F* = 7.423 (1,340) *p* = .007	.15
Factor 3: Social affirmation	0.052	–0.234	*F* = 4.19 (1,340) *p* = 0.041	.11

Examination of change over the 10 months of the project in the music groups (both novices and those who were more experienced) and those in the control groups revealed no statistically significant changes, positive or negative. This lack of change may reflect the relatively short period of time over which the research was undertaken or that participation in any group activity can contribute to the ongoing well-being of older people as might be expected.

Comparisons were made between those in the Third (50–75) and Fourth (over 75) age in the music groups. This was not possible for the control group as there were insufficient participants in the 4th age. This analysis revealed no differences in relation to factors relating to autonomy/control or social affirmation, although there was a deterioration in relation to sense of purpose. This suggested that the musical activities helped to prevent decline in autonomy/control and social affirmation which normally occur as individuals move into the 4th age.[15] The details are set out in Table 2.

**Table 2.** Differences in factor scores for 3rd and 4th age participants in the music groups.

	3rd age (*N* = 209)	4th age (*N* = 64)		Effect size
Factor 1: Purpose	0.157	–0.179	*F* = 5.819 (1,271) *p* = .017	.13
Factor 2: Autonomy/control	–0.107	0.032	*F* = .943 (1,271) NS	.06
Factor 3: Social affirmation	0.060	0.056	*F* = .001 (1,271) NS	.002

The questionnaires also asked participants to respond to a series of statements relating to the perceived benefits of group activity. High ratings were given by those participating in music and non-music groups to a series of statements relating to: sustaining well-being, quality of life and reducing stress; acquiring new skills; providing opportunities for mental activity and intellectual stimulation; promoting social activity and involvement in the community; providing opportunities for demonstrating skills and helping others; and maintaining physical health. There were no statistically significant differences in response to the elements outlined above between music and non-music groups. However, those participating in the music groups reported higher levels of enjoyment.[16,17]

### Interviews, focus groups and videos

Individual and focus group interviews with participants and facilitators revealed a range of perceived benefits of active musical engagement including those related to social activity, cognition, emotional and mental health and physical health. Social benefits included a sense of belonging, and a sense of playing a valued and vital role within a community. Participants also noted that being a member of a musical group helped to provide a routine and structure to their daily lives, providing motivation for leaving the house and for engaging in daily individual practice. The activities were also perceived as being fun and were recognised as a vehicle for respite for caregivers. Cognitive benefits included improved concentration, memory and meeting new challenges. The activities generated a sense of achievement particularly when rehearsals culminated in musical performances and the participants appreciated engaging in new activities. Participants also felt that they were making progress. Progression building on previous experiences and new learning played key roles in underpinning these benefits. Physical and health benefits included a renewed sense of vitality and rejuvenation and improved mobility. Emotional and mental health benefits were noted by participants and facilitators including reduction in stress and alleviation of depression. Participants specifically reported that the activities offered support following bereavement. Participants reported that engaging in the musical activities gave them a sense of purpose in life. There was also evidence of enhanced confidence in participants which in some cases were very marked. Participants commented on the specific role of music in enhancing their well-being. Overall, when questioned about what was special about music as opposed to other activities, many participants attributed positive benefits to the creative and expressive qualities of music.[18,19]

Opportunities for performance played a major role in the perceived benefits constituting a means of receiving position affirmation from others. For many participants, performances offered an important opportunity to ‘be a musician’, sharing the results of their hard work with friends and relatives. Performances were opportunities for positive feedback and contributed significantly to a strong musical self-concept, although it was important that participants perceived their contribution to be valued and meaningful.[20]

Some of the older people from the sheltered housing community engaged in intergenerational activities with children from local primary schools.[21] This energised them, was enjoyable and gave them an opportunity to relate to the younger generation. The intergenerational activity was an opportunity for different generations to socialise, show respect for each other and enjoy each other’s company. Teachers observed the sense of enjoyment and respect among the children, while the children described the experience as exciting, interesting and fun.

## Discussion

Overall, the findings from the Music for Life project supported that from the international literature. Those participating in musical activities had consistently higher levels of measured well-being that those participating in other group activities. These benefits continued from the Third Age into the Fourth Age. Participants reported social, cognitive, emotional and health benefits. As the fastest growing age cohort is represented by those over 85 years, there is a growing interest in activities which may promote active aging supporting a high quality of life, independence and competence. Music making clearly has the potential to achieve this.

The marked differences between the positive outlook of music and control cohorts may merely indicate that those who chose to engage with music (novices or those with more experience) already had a more positive outlook on life than those choosing to participate in other group activities. The interviews and focus groups suggested that engagement in music is a major contributing factor to a positive outlook, but this research did not have the power to establish the extent to which a positive outlook was required to engage in music in the first instance.

The research does not claim that music is the only social activity which can have a positive impact on health and well-being, but making music does seem to have a greater impact than other group activities. What is it about group music making activities that may contribute towards self-perceived well-being beyond participation in other social activities? Those in the comparison groups were engaged in a wide variety of activities including those focusing on physical activity, e.g. yoga classes; intellectual stimulation, e.g. language classes, book clubs; creative activities, e.g. art and craft classes and social activities, e.g. social support. Participation in music making provides stimulation in relation to all of these. In addition, group music making can support sense of purpose particularly when performances are planned. It also provides opportunities for having fun and deriving pleasure. Control and autonomy may be supported through: the physical activity involved in singing and playing instruments; the positive emotions that derive from music making which may impact to reduce depression; being challenged and given the opportunity to learn new skills and enhance already existing skills; and the nature of active music making which requires focus and concentration at a specific point in time which is not self-determined. Social affirmation may be supported through the opportunities provided for: social interaction; giving and receiving peer support; and performance which confers status, a sense of giving something back to the community, pride and opportunities for positive reinforcement.

In the research, a number of potential barriers to participation were identified. These included a lack of information about what was available locally. GP practices could assist in relation to this by making information about musical and other social activities available in their surgeries. They might also consider encouraging patients to engage in organised social activities including those involving active engagement in music not as an alternative to medical interventions but in addition to them.

## Disclosure statement

No potential conflict of interest was reported by the authors.

## Funding

This research was part of the New Dynamics of Aging programme which was funded across the five UK research councils: AHRC, BBSRC, EPSRC, ESRC, MRC.
